# Feasibility and Preliminary Results of a Multifamily Program for Children of Parents with Mental Illness

**DOI:** 10.1192/j.eurpsy.2026.10998

**Published:** 2026-07-31

**Authors:** O. Amiot, E. Petiau, D. Waintrater, L. Koziatek Molina, I. Fradi, M. C. Beaucousin

**Affiliations:** 93, Etablissement Public de Santé Ville Evrard, Saint Ouen, France

## Abstract

**Introduction:**

Children of parents with mental illness face elevated risks of emotional, social, and academic difficulties. Despite these vulnerabilities, adult psychiatric care rarely addresses the parenting role or children’s needs. Multifamily interventions such as the Kidstime model have shown promising results in several European countries, offering children explanations, reducing stigma, and strengthening parents’ confidence. To extend this approach in France, we developed UMPVE (*Un Moment Pour Vos Enfants*), a Kidstime-inspired program in our adult psychiatry department.

**Objectives:**

This pilot study aimed to explore the feasibility and acceptability of UMPVE and to describe the sociodemographic and clinical characteristics of the participating families.

**Methods:**

UMPVE is a multifamily psychoeducational program including eight monthly workshops for adults in psychiatric care, their partners, and children aged 5–18.

Sessions combine parallel groups for parents and children, creative play, shared discussions, and informal family time. Families were referred by their treating psychiatrist and attended a pre-admission interview. Nine families were included in the pilot group launched in December 2024.

Data collected included sociodemographic and clinical information, family composition, participation rates, and qualitative observations from families and facilitators.

**Results:**

Participants were mainly mothers (89%), often single (67%), unemployed (67%), and socially isolated (44%). Most were followed for mood disorders (56%) or psychotic disorders (22%), generally for less than three years, and 44% had never been hospitalized. Families included between one and four children. Three families discontinued participation after one or two sessions. Among those who continued, parents showed regular attendance (median 5 sessions, range 1–8). Children participated actively across sessions (median 4, range 1–8), while partner involvement was limited (4 partners attended, mostly once).

Observations highlighted children’s engagement in activities, parents’ reflection on their parenting role, and the restoration of parental self-esteem. The program was perceived as neither infantilizing nor judgmental, but as a supportive space fostering open dialogue and resilience.

**Conclusions:**

The pilot demonstrates the feasibility and acceptability of a Kidstime-inspired multifamily program within adult psychiatry. Despite socio-economic vulnerability and frequent isolation, families engaged actively and reported benefits in communication and parental confidence. These preliminary findings are consistent with international literature, underscoring the value of such programs as innovative, non-stigmatizing interventions that address a neglected public health issue. Further evaluation, including qualitative analyses, is warranted to better assess their impact on family dynamics and clinical outcomes.

**Disclosure of Interest:**

None Declared

